# An Evaluation of Interindividual Responses to the Orally Administered Neurotransmitter ****β****-Alanine

**DOI:** 10.1155/2013/429847

**Published:** 2013-06-23

**Authors:** Sarah MacPhee, Ian N. Weaver, Donald F. Weaver

**Affiliations:** ^1^Department of Medicine (Neurology), Dalhousie University, Halifax, NS, Canada B3M 3R4; ^2^Department of Biomedical Engineering, Dalhousie University, Halifax, NS, Canada B3M 3R4; ^3^Department of Chemistry, Dalhousie University, Halifax, NS, Canada B3M 3R4

## Abstract

Previously, we have identified **β**-alanine as a potential endogenous anticonvulsant molecule. **β**-Alanine occurs within the human central nervous system and has been identified as both an inhibitory neuromodulator and neurotransmitter that is bioavailable to brain after oral administration. During preliminary compounding trials to ascertain dosing strategies for **β**-alanine, we noted pronounced differences in the side effect profile experienced by individuals of Asian and Caucasian descent. To investigate whether ethnicity affects **β**-alanine-induced side effects, we administered 3 g of **β**-alanine in 200 mL of fruit drink to ten people of each ethnic background and observed them for 30 minutes. Data collected included basic physical statistics (height, age, and weight) and descriptions of all side effects, as reported by participants. We found that participants of Asian descent experienced paraesthesia, but significantly different in time of onset, intensity, and anatomical localization, as compared to the effects experienced by Caucasian participants. Since **β**-alanine is an endogenous neurotransmitter substance within human brain, these side effect differences were unexpected.

## 1. Introduction

Epilepsy is the most common serious chronic neurological brain disorder afflicting humankind, with no racial, socioeconomic, national, or geographic predilections; it affects more than 50 million people worldwide. The tendency of epilepsy to onset in children renders its socioeconomic impact disproportionate; moreover, the disorder is also associated with high comorbidity and significant life-long stigmatization. Since current anticonvulsant drugs are effective in less than 65% of people (and their use is associated with side effects in more than 50% of people), the need for new, effective, and safe therapies for epilepsy is a continuing neuropharmacological priority [1]. To address this need, we have sought to identify an “endogenous anticonvulsant molecule (ECM)” (i.e.,   the brain's own anti-seizure compound) as a platform around which to develop new drugs. Through this work, we have identified *β*-alanine as one possible candidate ECM [2, 3].

**Figure 1:**
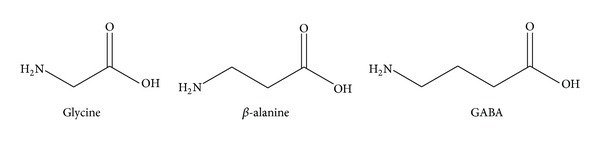
*β*-Alanine is a structural intermediate between glycine and GABA.

*β*-Alanine occurs within the human central nervous system (CNS) and has been identified as both an inhibitory neuromodulator [4] and neurotransmitter [5]. *β*-Alanine is structurally intermediate between *α*-amino acid (glycine) and *γ*-amino acid (GABA) inhibitory neurotransmitters (Figure 1). *β*-Alanine satisfies the prerequisite classical criteria for being a neurotransmitter: *β*-alanine occurs naturally in the CNS, is released by electrical stimulation through a Ca^2+^-dependent process, has binding sites, and inhibits neuronal excitability. *β*-Alanine has five recognized receptor sites: glycine coagonist site on the NMDA (N-methyl-D-aspartate) complex (strychnine-insensitive); glycine receptor site (strychnine sensitive); GABA-A receptor; GABA-C receptor; and blockade of GABA-transporter (GAT) protein-mediated glial GABA uptake. *β*-Alanine binding has been identified throughout the neocortex, hippocampus, and various limbic structures. Despite being a simple amino acid, *β*-alanine remains essentially unexplored as either a neurological drug or a drug design platform (although it has been used as a dietary supplement).

*β*-Alanine, found in both the CNS and skeletal muscle, has been studied extensively for its role in biosynthesis of the carnosine dipeptide [6]. It has been claimed that augmented levels of carnosine, achieved through *β*-alanine dietary supplementation, may enhance exercise performance and delay exhaustion by increasing the buffering capacity of skeletal muscle. Therefore, it has been suggested that the anaerobic working capacity of muscle tissue can be increased by ingesting *β*-alanine [7, 8]. In many countries, *β*-alanine is commercially available as a “muscle-building” dietary supplement and is used widely by males and females of varying ages, from young adults to seniors [9–12]. The typical daily dose averages 2–6 grams, administered orally, with the main side effects reported to be temporary sensory paraesthesia and mild flushing [13]. 

During initial toxicology studies to evaluate *β*-alanine as a putative orally active ECM, we anecdotally noted a pronounced difference in the side-effects experienced by individuals of Caucasian and Asian descent. Although individuals of Asian descent did experience some paraesthesia it was markedly delayed, milder, and presented initially in different locations than the paraesthesia experienced by Caucasian participants. Since *β*-alanine is an endogenous neurotransmitter substance within the human CNS (and is not an atypical or exogenous molecule), these differences were unexpected. Accordingly, we have pursued a pilot study to assess different interindividual physiological responses to the orally administered neurotransmitter *β*-alanine. Our study design has addressed this previously unreported finding, which could have implications for the clinical use of *β*-alanine as an ECM.

## 2. Materials and Methods

This investigator-initiated clinical trial was conducted at a single study centre, without blinding, randomization, or crossover. The trial was designed to test the hypothesis that after 30 minutes individuals of Asian descent experience different and/or delayed side-effects that are milder in nature and have a different pattern of distribution, from those observed in individuals of Caucasian descent. For the purposes of this study we define Asian descent to mean that both parents are of oriental heritage, including origins in South Asia, Southeast Asia, or East Asia. We define Caucasian descent to mean that both parents are white individuals, regardless of heritage. Secondary endpoints include the subjective side-effect profile described by study participants, aged 18 years and older.

Ten adult volunteers of each ethnicity were recruited and upon providing informed consent were given a single 3 g dose of *β*-alanine, purchased commercially from Allmax Nutrition and verified for its purity and composition. The study drug was dissolved in 200 mL of an artificial fruit-flavoured drink (Tang), which participants were instructed to drink completely over a timed four-minute oral consumption period.

Participants remained for 30 minutes after taking the study drug for observation and discussion detailing any side-effects they experienced. After 30 minutes, participants were asked to complete a questionnaire to score their perception of the symptoms they experienced upon ingesting *β*-alanine from 1 (no symptoms) to 5 (extremely unpleasant symptoms). The questionnaire was comprised of nine “symptom questions,” which sequentially asked responders whether they experienced pins and needles (paraesthesia) and/or itching around their mouth, ears, arms, hands, or legs and, finally, whether or not they felt themselves to be flushing. These nine questions are given in Table 1. The demographic information was aggregated together and analyzed for trends among interpersonal variables.

All statistical comparisons were two tailed, with *P* values < 0.05 considered significant. Analysis was based on intention to treat, comparing change from baseline in measures of symptoms between the two treatment groups by means of *t*-tests. Characteristics were tested for significant differences between the two treatment groups by means of two-tailed paired *t*-tests. 

## 3. Results and Discussion

Data were analyzed to address the hypothesis whether inherent differences exist between Asians and Caucasians in terms of reported side-effects following oral administration of *β*-alanine.

The average age of both Asian and Caucasian participants was 31 years. In both groups, the male/female ratio was 7/3. The average weight of Caucasians, at 170 pounds, was greater than in the average weight of Asians, at 139 pounds; however, the Body Mass Index (BMI) was 24.9 for Caucasians and 22.2 for Asians (not statistically significant difference).

**Figure 2:**
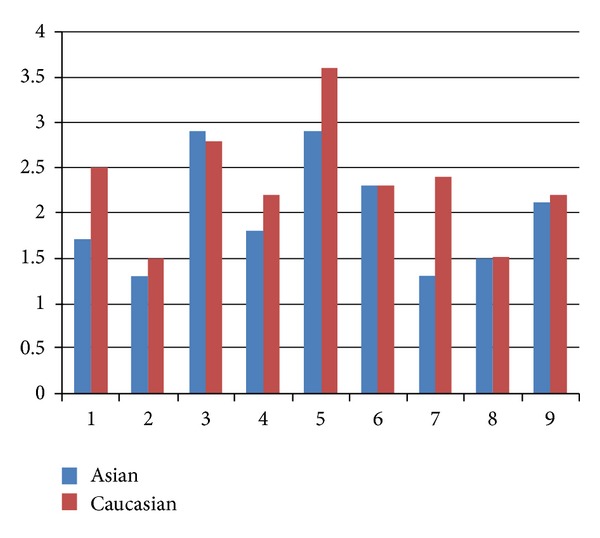
Average responses to questions 1–9 in Asians versus Caucasians.

We compared the average and median responses to each of the nine questions. Average responses in terms of perceived side-effect severity were lower in Asians than Caucasians in 6/9 instances, as shown in Figure 2. Asians self-reported their symptoms to be substantially less unpleasant than Caucasians. Caucasian participants reported an average of 5.1 symptoms in the 30-minute observation period, while Asians reported an average of only 2.2. (This could speak to personal attributes of participants; that is, certain individuals are more willing to freely describe everything they experience, no matter how minor and considering many of the Asian participants were communicating predominately in their second language (English) this may have been a hindrance to free and open communication. Accordingly, a translator was available during data collection to minimize any language barrier issues.)

When the results of all nine symptom questions are summed, there is a statistically significant difference between Asians and Caucasians (*P* < 0.05). However, when each of the individual symptom questions is independently analyzed, only two demonstrate statistical significance (*P* < 0.05): (i) pins and needles sensation in arms/hands and (ii) pins and needles sensation in legs and feet. These latter two symptoms were much more marked in Caucasian subjects versus Asian subject.

Also evident when examining participant's self-reported symptoms is that while Caucasians averaged 8 minutes prior to their first reported symptom, Asians experienced their first symptoms after 14 minutes or longer had elapsed; furthermore, the onset of symptoms in Caucasians was acute and relatively intense whereas symptom onset in Asians was gradual and with low initial intensity. Therefore, participants of Asian descent had an average of six further symptoms free minutes prior to experiencing anything that differed from their baseline. Additionally, there was a tendency for Asians to report that they felt their first symptom in a unique bilateral retro-aural distribution (over the mastoid process, posterior to the external pinna of the ear) while Caucasians reported to notice their first symptoms within an extremity (particularly the anterior upper thigh).

**Figure 3:**
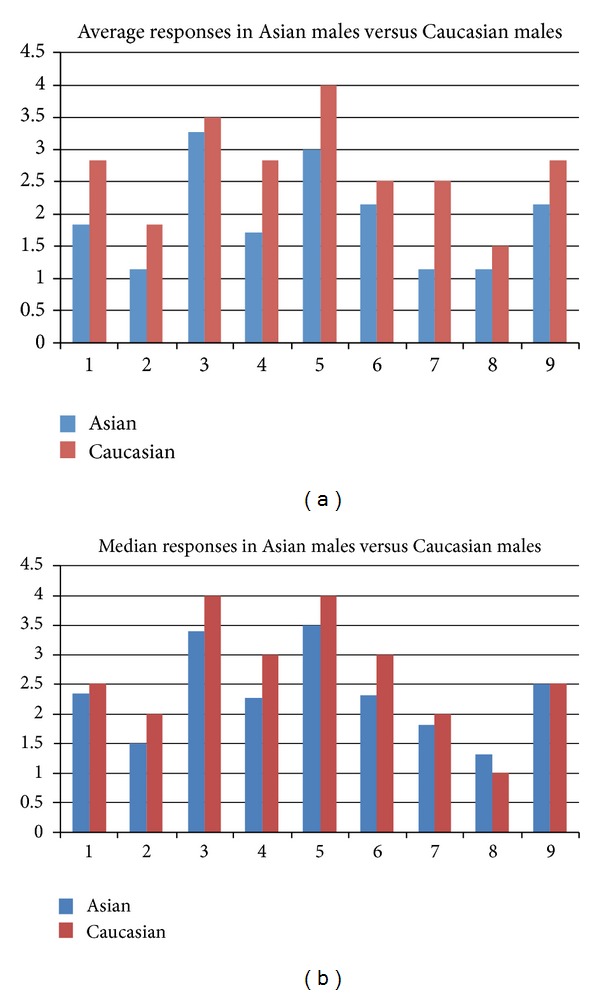
Average and median responses in asian males versus caucasian males.

Subsequently, we examined whether participant gender was contributing to data trends, rather than ethnicity alone. By considering the average and median responses to the questionnaire by Asian males versus Caucasian males and Asian females versus Caucasian females; there was clear trend that was evident amongst male participants. For every question, Caucasian males ranked their symptoms to be more severe than their Asian counterparts. The same was true for the median responses in 8/9 instances, as represented by Figure 3.

**Figure 4:**
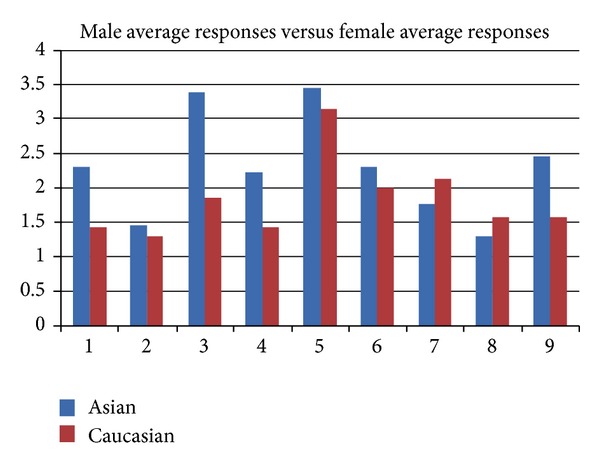
Average responses in males versus females.

Asian females reported their symptoms to be more bothersome than Caucasian females (the opposite of what was true in males) and were relatively equal in reported severity when considering median answers. However, regardless of ethnicity, males seemed to be more bothered by their symptoms than females, see Figure 4.

The fact that Caucasian males reported more frequent and more intense side-effects seems peculiar as an increase in side-effects is expected with lower body mass, as it is the case with most pharmaceuticals. However, we noted the opposite: with Caucasian males experiencing the most side-effects, yet having the highest body weight of participants. Future investigation may be required with increased number of participants to explain the observed variances between ethnicities and to understand whether the mechanism of action is the same and what this means in terms of the interindividual efficacy of *β*-alanine. Future clinical research investigating *β*-alanine will have to be sensitive to the fact that the side-effect profile can vary based on gender and ethnicity. This notion will have to be given careful thought considering the challenges of blinding a study drug that produces notable and distinctive side-effects within 30 minutes of ingestion.

## 4. Conclusions

In conclusion, following oral administration of the *β*-alanine neurotransmitter, there were definite differences in the timing, localization, and intensity of side-effect perception between Asians and Caucasians. Asian participants had an average of six more symptom-free minutes prior to experiencing any side-effects. Although participants of Asian descent did experience paraesthesia, they were delayed with different initial side-effects and distribution, when compared to the effects experienced by Caucasian participants. When Asian participants did report an initial side-effect, it was predominately localized in a retro-aural distribution. Lastly, we observed a statistically significant higher prevalence of pins and needles sensation in the upper and lower extremities in Caucasians.

This pilot study challenges the assumption that an amino acid with properties of a neurotransmitter should have similar side-effects in everyone, regardless of ethnicity. Although racial and ethnic differences in response to synthetic drug molecules are well known [14], *β*-alanine is an endogenous neurotransmitter substance within the human CNS (and is not an atypical or exogenous molecule), and thus these differences were unexpected. This study also foreshadows challenges in running a “blinded” clinical trial assessing the efficacy of *β*-alanine versus a placebo in the treatment of epilepsy (since participants receiving *β*-alanine will experience a characteristic set of paraesthesia side-effects).

## Figures and Tables

**Table 1:** Nine symptom questions administered to participants after the elapse of 30 minutes.

	Not bothersome	Somewhat bothersome			Extremely bothersome
(1) Pin and needles around mouth	1	2	3	4	5
(2) Itching around mouth (circumoral)	1	2	3	4	5
(3) Pins and needles around ears	1	2	3	4	5
(4) Itching around ears	1	2	3	4	5
(5) Pins and needles in arms and/or hands	1	2	3	4	5
(6) Itching in arms and/or hands	1	2	3	4	5
(7) Pins and needles in legs	1	2	3	4	5
(8) Itching in legs	1	2	3	4	5
(9) Flushing (reddening and/or feeling of heat)	1	2	3	4	5
